# Two-to-three-year effectiveness and safety of nurse-led accelerated corneal crosslinking for progressive keratoconus: The Birmingham and Midland Eye Centre Study

**DOI:** 10.1038/s44440-026-00013-z

**Published:** 2026-02-09

**Authors:** Murad Khan, Rashvinder Sahota, Joan Hanson, Yasmin Cabdi, Marianthi Bourlaki, Catey Bunce, Anil Aralikatti, Ankur Barua, Darren S. J. Ting

**Affiliations:** 1 Birmingham and Midland Eye Centre, Sandwell and West Birmingham NHS Trust, Birmingham, UK; 2 Birmingham Medical School, University of Birmingham, Birmingham, UK; 3 London School of Hygiene & Tropical Medicine, London, UK; 4 Academic Unit of Ophthalmology, Department of Inflammation and Ageing, College of Medicine and Health, University of Birmingham, Birmingham, UK; 5 Academic Ophthalmology, School of Medicine, University of Nottingham, Nottingham, UK

**Keywords:** Corneal diseases, Health services

## Abstract

**Background/Objectives:**

To examine the two-to-three-year effectiveness and safety of accelerated corneal crosslinking (CXL) treatment performed by ophthalmic nurses for progressive keratoconus and to evaluate the potential prognostic factors for disease progression.

**Methods:**

All consecutive eligible patients with progressive keratoconus who underwent nurse-led CXL between February 2019 and December 2021 at the Birmingham and Midland Eye Centre, UK, were included. A standardised accelerated, epithelium-off CXL protocol was employed, using 10 mW/cm^2^ ultraviolet-A irradiation for 9 min (5.4 J/cm^2^). Relevant data, including demographics, corrected-distance-visual-acuity (CDVA), corneal tomographic findings, outcomes and adverse event, were analysed. Only patients that had completed at least a 2-year follow-up post-CXL were included.

**Results:**

We included 97 patients (*n* = 97 eyes); mean age was 26.4±6.5 years and 52.6% were female. Most patients had mild (stage I) keratoconus (49.5%). From baseline to 24-month post-CXL (*n* = 97 eyes), there was a significant improvement in CDVA, (0.30 ± 0.22 logMAR vs. 0.20±0.18 logMAR; *p* < 0.001) and Kmax (59.1±6.8 D vs. 58.1±6.4 D; *p* < 0.001), with similar K1, K2 and thinnest corneal pachymetry (all *p* > 0.05). There were three (3.1%) cases of clinically significant transient corneal haze noted at 1-week post-CXL. No significant adverse events such as corneal infection/melting were observed. At 36-month post-CXL (*n* = 31 eyes), the CDVA remained stable, with a significant improvement in Kmax (*p* < 0.001) and K2 measurements (*p* = 0.038) from baseline.

**Conclusions:**

This study highlights the efficacy, safety and feasibility of nurse-led CXL, serving as a valuable initiative in addressing the increased service demand for keratoconus management and preserving the vision of patients with keratoconus.

## Introduction

Keratoconus is the most common corneal ectasia worldwide, with an estimated prevalence of 0.5–3 per 1000 population [1]. It is a progressive corneal condition characterised by corneal thinning and steepening, resulting in irregular astigmatism and myopia [1, 2]. Early diagnosis and timely intervention is vital to stabilise the condition, preserve vision, and decrease the potential need for corneal transplantation [1, 2].

Over the past two decades, there has been a paradigm shift in the management of keratoconus. Depending on the severity and progressiveness of the condition, the management encompasses conservative treatment (e.g., spectacles and soft/rigid contact lenses), corneal crosslinking (CXL) and CXL-plus, intracorneal ring segments, anterior lamellar and penetrating keratoplasty, Bowman’s layer transplantation, stromal keratophakia, and stromal regeneration. Although only a limited number of patients require keratoplasty for visual improvement, keratoconus remains one of the leading indications for corneal transplant in many regions [3, 4].

CXL was first introduced in 2003 by Wollensak et al. to stabilise progressive keratoconus [5]. This procedure aims to strengthen the corneal biomechanical rigidity and stability through the use of ultraviolet-A (UVA) light of 370 nm and topical riboflavin drops. Currently, it is considered the only treatment option that addresses the underlying pathophysiology and halts the progression of keratoconus. Since the original introduction of the epithelium-off Dresden protocol (i.e. 30 min of topical riboflavin 0.1% drops, followed by 3 mW/cm^2^ UVA over 30 min; 5.4 J/cm^2^), several protocol variations have been reported and implemented, including accelerated (e.g. 10 mins of 9 mW/cm^2^ UVA, 3 mins of 30 mW/cm^2^), epithelium-on / transepithelial, contact lens-assisted, and adapted fluence protocols, to improve treatment efficiency and safety [6–10]. Long-term studies have supported the effectiveness and safety of CXL in stabilising keratoconus progression [11, 12]. In addition, several studies have demonstrated a reduction in the number for keratoplasty required for keratoconus following the introduction of CXL, highlighting its important role in the management of keratoconus [13, 14].

Over the recent years, there has been a considerable rise in community-to-hospital referrals for keratoconus management in the UK, leading to increased demand on hospital services and longer waiting time for patients to be reviewed and managed. Upskilling ophthalmic healthcare practitioners (OHPs) has been identified by The Royal College of Ophthalmologists (RCOphth) as a way to meet rising demand on healthcare services [15]. For example, several nurse-led and optometrist-led keratoconus services have been established to assess and manage keratoconus (which includes performing CXL), though long-term evidence is lacking [16, 17]. In view of the gap in the literature, our study aimed to examine the effectiveness and safety of the nurse-led CXL service over a 24-to-36-month period and to examine the prognostic factors for post-CXL progression.

## Materials and methods

This study was approved by the clinical governance team of Sandwell and West Birmingham Hospitals NHS Trust as a clinical audit (Ref: 1617). Patients who underwent nurse-led CXL [all performed by a trained specialist nurse (RS)] between February 2019 and December 2021 were included initially. Informed consent was obtained from all patients prior to the CXL treatment. A standardised accelerated, epithelium-off CXL protocol was used, with 10 mW/cm^2^ UVA irradiation for 9 min (5.4 J/cm^2^). The ophthalmic nurse performing the procedure was initially supervised by a consultant ophthalmologist for 20 consecutive cases before performing the procedure independently thereafter. Relevant data, including demographic factors, past ocular and medical history, CDVA (in logMAR), corneal tomographic findings, outcomes and adverse event (if any), were collected from the local electronic patient records system and the Scheimpflug camera and its associated software (OCULUS Pentacam®, Wetzlar, Germany). Severity of keratoconus was graded based on Amsler-Krumeich classification [1]. Only those who had completed at least a 2-year follow-up post-CXL within our hospital were included in our study. Only the right eye was included for bilateral CXL cases. Progression of keratoconus was defined by the presence of ≥2 following criteria over the past year [3]:

- Subjective/objective decrease in CDVA ≥ 1 logMAR line
- ≥1D increase in cylinder on manifest refraction
- ≥1D increase in K2
- ≥1D increase in Kmax
- Progressive corneal thinning

Belin-Ambrosio Enhanced Ectasia Display was used to aid the diagnosis in borderline cases. This is a display available in OCULUS Pentacam software that combines anterior and posterior elevation data, pachymetry progression, and thinnest corneal point location and measurement. This produces a deviation score that quantifies deviation from normal healthy corneal structure. Amsler-Krumeich classification was used to grade the severity of keratoconus: (1) stage 1—Kmean of <48 dioptres (D) and thinnest corneal pachymetry (TCP) > 400 microns; (2) stage 2—Kmean of <53 D and/or TCP > 400 microns; (3) stage 3—Kmean of > 53 D and/or TCP of 300–400 microns, and (4) stage 4—Kmean > 55 D, TCP of 200 microns, and/or central corneal scarring.

The patients were initially reviewed at every 3–4 months after their first clinic visit during the first year and at every 6–9 monthly thereafter at the clinicians’ discretion. Poor visual outcome was defined as CDVA ≤ 0.3 logMAR (or ≤ 6/12 Snellen vision) at 24 months following CXL. Significant post-CXL progression of keratoconus was defined as >1 D increase in Kmax from preoperative to 24 months following CXL.

### Surgical technique

All cases were done using an operating microscope and aseptic technique under topical local anaesthesia as day surgery. Briefly, topical oxybuprocaine hydrochloride 0.4% was instilled before the start of the procedure. The skin around the eye was prepared with povidone-iodine 5%. After applying a sterile drape and inserting a lid speculum, further topical oxybuprocaine hydrochloride 0.4% was administered. The central 8 mm cornea was then removed by a hockey stick (incorporating the Fleischer ring, if present, to fully capture the cone in order to effectively stabilise the disease). Topical 0.1% riboflavin drops with 1.1% hydroxypropyl methylcellulose without dextran (MedioCROSS M isotonic, Medio-Haus Medizinprodukte GmbH, Germany) were instilled onto the cornea every 1–2 min for 15 min. UVA (365 nm) irradiation with an energy release of 10 mW/cm^2^ for 9 min (fluence of 5.4 J/cm2) was then applied using the VEGA CBM-X-Linker (CSO, Firenzi, Italy).

At the end of the procedure, topical preservative-free chloramphenicol 0.5%, oxybuprocaine hydrochloride 0.4%, and cyclopentolate hydrochloride 1% were instilled in the eye. Postoperative treatment regime included topical preservative-free carmellose 1% drops 6 times a day for 1 month, cyclopentolate hydrochloride 1% drops 3 times a day for 3 days, chloramphenicol 0.5% drops 4 times a day for 7 days, and oral co-codamol 30/500 mg 1–2 tablets QID as required for 3 days. Topical preservative-free dexamethasone 0.1% drops were added at 1-week postoperative follow-up visit when the cornea was fully epithelialised.

### Statistical analysis

CDVA was analysed in logMAR, with 0.0 and 1.0 being equivalent to Snellen vision of 6/6 and 6/60, respectively. Continuous values are presented as mean ± standard deviation (SD). Student’s *t* test (paired and unpaired) and Fisher exact test / Chi square test were used for analysis of the mean difference and categorical variables between two groups, respectively. Multivariate logistic regression was performed to examine the potential prognostic factors of post-CXL progression, defined by an increase in Kmax by 1 D at 2-year post-CXL. *P* value of <0.05 was considered statistically significant.

## Results

During the study period, a total of 204 patients underwent nurse-led CXL. Of these, 97 patients (*n* = 97 eyes) completed 24-month follow up, and 31 patients (*n* = 31 eyes) completed 36-month follow-up. The patient’s mean age was 26.4 ± 6.5 years and 52.6% were female (Table 1). Based on Amsler-Krumeich classification, 48 (49.5%), 31 (32.0%), 8 (8.2%), and 10 (10.3%) had stage 1, 2, 3, and 4 keratoconus, respectively. The majority of the patients had a presenting CDVA of ≤0.3 logMAR.

**Table 1:** Summary of baseline characteristics of 97 patients (*n* = 97 eyes) with progressive keratoconus in Birmingham and Midland Eye Centre, United Kingdom.

Parameters	*N* (%) or Mean (SD)
Age, years	26.4 (6.5)
Gender	
Female	51 (52.6)
Male	46 (47.4)
Laterality	
Right eye	56 (57.7)
Left eye	41 (42.3)
Presenting CDVA, logMAR	0.30 (0.22)
≤0.3	74 (76.3)
>0.3 to 0.6	14 (14.4)
>0.6	9 (9.3)
Severity of keratoconus*	
Stage 1	48 (49.5)
Stage 2	31 (32.0)
Stage 3	8 (8.2)
Stage 4	10 (10.3)
Corneal Tomographic Findings	
Kmax, D	59.1 (6.8)
K1, D	47.3 (4.2)
K2, D	50.7 (4.8)
TCP, μm	454.8 (38.4)

^*^Severity is based on Amsler-Krumeich classification.

*CDVA* corrected-distance-visual-acuity, *D* dioptres, *Kmax* maximum keratometric reading, *K1* flattest keratometric meridian, *K2* steepest keratometric meridian, *SD* standard deviation, *TCP* thinnest corneal pachymetry.

From baseline to 24-month post-CXL, there was a significant improvement in CDVA (0.30 ± 0.22 logMAR vs. 0.20±0.18 logMAR; *p* < 0.001) and Kmax (59. 1 ± 6.8 D vs. 58.1 ± 6.4 D; *p* < 0.001), with similar K1, K2 and thinnest corneal pachymetry (all *p* > 0.05; Table 2). The proportion of patients having CDVA of ≤0.30 logMAR increased from 74 (76.3%) to 86 (88.7%) patients (*p* = 0.023). There were three (3.1%) cases of clinically significant, transient corneal haze noted at 1-week post-CXL, which resolved quickly with postoperative topical steroids within a month post-CXL. No patient experienced >2 lines loss of CDVA noted at 24-month post-CXL. No significant adverse events such as corneal infection or melting were observed.

**Table 2:** Summary of visual and corneal tomographic outcomes of 97 patients (*n* = 97 eyes) with keratoconus at baseline, 12 months, and at 24 months following nurse-led corneal crosslinking (CXL).

Parameters	Baseline	12 months	*P*-value*	24 months	*P* value*
CDVA, logMAR**	0.30 (0.22)	0.27 (0.25)*	0.28	0.20 (0.18)	<0.001
**Kmax, D**	59.1 (6.8)	58.6 (6.6)	0.02	58.1 (6.4)	<0.001
**K1, D**	47.3 (4.2)	47.4 (4.4)	0.31	47.5 (4.4)	0.11
**K2, D**	50.7 (4.8)	50.8 (4.6)	0.85	50.6 (4.5)	0.18
**TCP, μm**	454.8 (38.4)	446.3 (48.9)	0.015	452.4 (37.9)	0.11

Values are presented in mean (standard deviation).

*Paired *t* test was used for comparing the findings between the baseline and 12 months or between baseline and 24 months post-CXL.

**BCVA results were missing on 14 subjects (at 12 months).

*CDVA* corrected-distance-visual-acuity, *D* dioptres, *Kmax* maximum keratometric reading, *K1* flattest keratometric meridian, *K2* steepest keratometric meridian, *TCP* thinnest corneal pachymetry.

At 24 months post-operative follow-up, 12 (12.4%) eyes were noted to have post-CXL progression (based on the study definition), but none required repeat CXL due to stability of vision. Multivariate logistical regression was performed to examine for potential prognostic factors post-CXL treatment. However, none of the examined factors, including age, gender, ethnicity, presenting CDVA, and tomographic parameters, were shown to be significant (all *P* > 0.05; Table 3).

**Table 3:** Prognostic factors of progression in keratoconus at 24 months post-corneal cross-linking (CXL).

Parameters	Odd ratio	*P* value*
Age (per year)	0.91 (0.81 to 1.02)	0.12
Male gender	1.65 (0.48 to 5.62)	0.42
Ethnicity		
Non-White	1 (reference)	-
White	0.57 (0.07 to 4.96)	0.61
Not stated	0.32 (0.04 to 2.64)	0.29
CDVA (per logMAR)	0.67 (0.04 to 11.22)	0.78
Kmax (per D)	0.96 (0.87 to 1.06)	0.44
K1 (per D)	0.95 (0.81 to 1.11)	0.49
K2 (per D)	0.99 (0.88 to 1.13)	0.93
TCP (per μm)	1.00 (0.99 to 1.02)	0.87

*Multivariable logistic regression analysis was performed.

*CDVA* corrected-distance-visual-acuity, *D* dioptres, *Kmax* maximum keratometric reading, *K1* flattest keratometric meridian, *K2* steepest keratometric meridian, *SD* standard deviation, *TCP* thinnest corneal pachymetry.

Of the 31 patients who completed the 36-month follow-up, there was a statistically significant improvement in Kmax (60.6 ± 6.4 D vs. 59.2 ± 7.2 D; *p* < 0.001) and K2 measurements (52.1 ± 4.4 D vs 51.6±4.7 D; *p* = 0.038) from baseline to 36-month postoperative (Table 4). CDVA, K1, and TCP measurements were similar between baseline and 36-month post-CXL.

**Table 4:** Summary of visual and corneal tomographic outcomes of 31 patients with keratoconus at baseline, 12 months, 24 months and at 36 months following nurse-led corneal crosslinking (CXL).

Parameters	Baseline	12 months	*P* value*	24 months	*P* value*	36 months	*P* value*
**CDVA, logMAR**	0.31 (0.25)	0.29 (0.27)**	0.82	0.25 (0.18)	0.08	0.26 (0.18)	0.15
**Kmax, D**	60.6 (6.4)	59.7 (7.4)	0.020	59.3 (7.1)	<0.001	59.2 (7.2)	<0.001
**K1, D**	48.3 (3.5)	48.1 (3.9)	0.35	48.4 (3.8)	0.69	48.3 (3.8)	0.93
**K2, D**	52.1 (4.3)	51.9 (4.6)	0.41	51.7 (4.6)	0.07	51.6 (4.6)	0.038
**TCP, μm**	443.0 (36.4)	431.1 (58.9)	0.20	439.7 (36.7)	0.20	440.9 (37.3)	0.47

Values are presented in mean (standard deviation).

*Paired *t* test was used for comparing the findings between the baseline and several post-CXL time points (including 12, 24, and 36 months).

**CDVA was missing on 6 participants.

*CDVA* corrected-distance-visual-acuity, *D* dioptres, *Kmax* maximum keratometric reading, *K1* flattest keratometric meridian, *K2* steepest keratometric meridian, *TCP* thinnest corneal pachymetry.

## Discussion

In this study, we highlight the efficacy and safety of a standardised, accelerated, epithelium-off CXL protocol, administered by a trained corneal specialist nurse, for treating progressive keratoconus over a 24-to-36-month period. Although the roles of OHPs, including nurses and optometrists, in keratoconus have been explored in a supportive capacity, there is limited literature evaluating their role in performing CXL. Prajapati et al. have recently published the results of their nurse-led CXL service, demonstrating good outcomes and safety over 1-year follow-up period [17]. A 2021 report from New Zealand also highlighted the value of a nurse-led CXL system and how this helped reduce the workload for ophthalmologists [18].

Following the first use of CXL in 2003 by Wollensak et al, there have been a number of studies that have explored the use of so-called accelerated protocols [5]. The concept of accelerated protocols is based on the Bunsen-Roscoe law of photochemical reciprocity, where the total energy remains unchanged if the irradiation duration is reduced with a corresponding increase in the intensity of irradiation [19]. The use of an accelerated protocol allows for the shortening of the irradiation treatment time while maintaining the same fluence of 5.4 J/cm^2^. Schumacher et al. noted that there was an equivalence in the corneal stiffening effect between the Dresden CXL protocol (3 mW/cm^2^ for 30 min) and the accelerated protocol (10 mW/cm^2^ for 9 min) using animal models [20]. Since then, many studies have investigated and compared the outcomes between accelerated and conventional protocols [1, 8, 11, 21, 22].

In a randomised controlled trial (RCT), Shetty et al. noted that there was a statistically significant improvement in terms of corneal flattening and CDVA improvement in protocols that used conventional (3 mW/cm^2^ for 30 min), and accelerated protocols (9 mW/cm^2^ for 10 min and 18 mW/cm^2^ for 5 min), with the latter group showing the best CDVA improvements overall [23]. Interestingly, the group that underwent CXL using 30 mW/cm^2^ for 3 min in this study did show an improvement in mean CDVA following 12 months, but this was not statistically significant, potentially highlighting an optimal level of intensity of CXL treatment. The suboptimal results observed for the more intense protocol of 30 mW/cm^2^ is in line with other studies assessing intensity of treatment in CXL [21]. A meta-analysis of 6 RCTs demonstrated similar effectiveness and safety between accelerated and conventional protocols in terms of visual acuity, keratometry, corneal biomechanical properties, and endothelial cell density [8]. Similarly, a recent comparative study showed that both accelerated and conventional CXL protocols were effective and safe in stabilising keratoconus, though the standard protocol resulted in greater improvements in vision and keratometry [11].

To date there are no studies in the literature reporting the long-term outcome of CXL performed by OHPs. Prajapati et al. reported the 12-month outcomes of 186 patients who underwent accelerated CXL performed by OHPs [17]. In their study, 46.3% of the patients experienced visual improvement, with no one lost ≥2 lines of vision. However, specific quantification of the level of vision improvement was not reported in their study. In our study we saw a significant improvement of mean CDVA (by -0.10 logMAR) at 24 months post-CXL, with a significantly higher number of patients achieving CDVA of ≤0.30 logMAR (or ≥6/12 Snellen vision) at 24 months post-CXL. This result was better or at least comparable with the findings reported by Ting et al. and Vounotrypidis et al., who observed a significant improvement in CDVA (by –0.05 logMAR) at 24–36 months following CXL performed by ophthalmologists, providing supportive evidence on the outcomes of nurse-led CXL [10, 22].

In addition to the significant difference shown in CDVA, our study observed an improvement in Kmax (by –1.0 D) at 24 months post-CXL. Kmax has been observed to stabilise or improve in several long-term studies in both conventional and accelerated CXL protocols, with approximately improvement of Kmax by 0.2–2.0 D [21, 22, 24–26]. We also observed a significant decrease in the TCP (by –8.5 microns or ~2%) at 12 months follow-up, but increased back to the preoperative level at 24 months follow-up. This was similar to other studies, which reported a significant decrease in TCP at 12 months follow-up and remained stable thereafter [10, 24].

Several studies have examined the prognostic factors of progression of keratoconus following CXL. For instance, Ting et al. previously noted a higher chance of post-CXL progression in patients with more severe keratoconus (i.e., greater Kmax and Kmean) at baseline [10]. On the other hand, Greenstein et al. noted that eyes with a poorer preoperative CDVA (0.30 logMAR or worse) and higher Kmax value (≥55.0D) were most likely to show visual improvement and corneal flattening at 12 months post-CXL [27]. In this study, we examined several potential prognostic factors of post-CXL progression (defined by >1D increase in Kmax at 24 months post-CXL), though none of the factors were shown to be significant. The lack of significant results might be attributed to the relatively small sample size of patients with postoperative progression at 24 months (*n* = 12), resulting in a potential type 2 error.

The long-term safety of both conventional and accelerated CXL is well established in many studies, however there is a lack of literature regarding CXL performed by OHPs. In our study, we noted three (2.7%) cases of mild corneal haze at 1-week post-CXL, which resolved quickly with post-operative topical steroid, and no significant adverse events such as corneal infection or melting were observed. Overall, our results were similar to the findings of other studies where CXL was performed by ophthalmologists [10, 21, 22, 24].

One of our study limitations was that the included cases might be subjected to a selection bias as these cases were pre-assessed by the consultants and the OHPs, ensuring that all cases were suitable for nurse-led CXL. Nevertheless, our patient demographics and disease severity were comparable to other comparative studies, where stage 1–4 keratoconus were included in this study. In addition, it would be interesting to compare the effectiveness and safety of CXL performed by ophthalmologists and OHPs.

In summary, our study demonstrated the two-year effectiveness and safety of nurse-led CXL for progressive keratoconus. In view of the encouraging results, we recommend the training and adoption of OHP-led CXL services within the UK and beyond. Further studies with larger sample size and longer follow-up would be useful to establish the long-term effectiveness and safety of nurse-led CXL in the future.

## Summary

### What was known before

- Corneal crosslinking (CXL) serves as an effective and safe treatment for stabilising progressive keratoconus.
- Timely treatment is essential for preserving good vision in keratoconus; however, current prolonged waiting time may adversely affect the visual outcome.
- The long-term effectiveness and safety of CXL performed by doctors has been well established, but long-term evidence related to nurse-led CXL is lacking.

### What this study adds

- This study highlights the three-year effectiveness and safety of nurse-led accelerated CXL treatment in stabilising progressive keratoconus.
- Ophthalmic healthcare professionals, including nurses, play a key role in delivering ophthalmic services and mitigating the increasing service burden faced by ophthalmology.

## Data Availability

All data generated or analysed during this study are included in this published article [and its supplementary information files].
